# The Vermiform Appendix and Appendicitis

**Published:** 1901-11

**Authors:** A. A. Young

**Affiliations:** Newark, N. Y.


### The Vermiform Appendix and Appendicitis.

By A. A. YOUNG, M. D., Newark, N. Y.

IT IS my purpose in this paper on the appendix and appendicitis, to present a few thoughts upon this most important subject, from the standpoint of a country practitioner, who must meet and treat these cases without the aid of an expert or the advantages of an emergency hospital. In country practice, oftentimes the fine spun theories of the expert must be brushed away like the webs of the spider by the hands of emergency.

1. Read at the thirty-fourth annual meeting of the Medical Association of Central New York, held at Buffalo, N. Y., August 27 and 28, 1901.

If in this paper, views are expressed which are antagonistic to those usually advanced, let me remind you that amongst chaff some wheat may be found. A brief anatomical review of the appendix and its environment will serve as an introduction.

I will begin by observing that the vermiform appendix is a worm-like process attached to the cecum near its lower and posterior border, at which point the cecum and appendix communicate by means of an opening which is guarded in some degree by a narrowing of the tube near the point of attachment and also by a peculiar valvular arrangement at this orifice. The appendix, from its attachment to the cecum, passes downward and inward toward the median line of the body; it is curved upon itself, which curvature is apparently produced by thickening and increased density of its wall upon the concave side.

The appendix is composed of a tough, thick and elastic membrane, a portion of which is made up of involuntary muscular fibers similar to those found in other portions of the intestinal tract. The whole organ also is usually enclosed in a fold of the peritoneum, which serves as a protection to it and an aid in the performance of its function. The normal substance found within the appendix is an exudate coming from the solitary glands which are an integral part of the organ itself. This exudate, when vitiated, doubtless becomes a leading factor in the development of appendicitis.

The cecum, to which the appendix is attached and of which it forms a part, is made up in a similar manner with muscular fibers and solitary glands having similar functions. This organ upon its inferior surface is blind, save as it communicates with the appendix, while its upper border joins upon and communicates with the ascending colon. It will also be observed that the opening of the cecum into the colon is looking upward, and at this juncture the ileum enters and discharges its contents more or less digested into the cecum. Along the sides of the cecum are found the beginnings of fibrous bands which extend upward and are continuous along the entire length of the colon on either side. These bands act as shir-strings to the colon, forming little pockets or sacs, the apparent use of which is to carry along the intestinal contents toward their point of exit. Such function is clearly indicated by the appearance of the normal feces when ejected from the body. The fecal mass as you have observed, contains markings, corresponding to these pockets upon its surface, or the feces may be composed of little masses corresponding to each fold, either adherent or nonadherent. These masses and markings clearly indicate what function each pocket performs—namely, that of catching the disintegrating fecal mass which is forced upward from the cecum by the normal action of the appendix, aided by the action of the cecum itself, and carrying such masses along the course of the colon to the rectum. It seems but reasonable to infer that the tendency to disintegration of the fecal mass within the cecum must depend largely upon its contact and admixture with the normal excreta coming from the solitary glands, which are found adjacent to the fecal mass and within the appendix.

Considering the anatomy of the appendix, inferentially, it has at least two functions—namely:

First.—To furnish a fluid which is an important element in the process of digestion, either mechanically as a lubricant or chemico-vitally as a digestant and solvent.

Second.—It acts as a reservoir for the accumulating gas in and about the cecum, which gas when accumulated in sufficient quantities distends the appendix and stimulates it to forcible contraction, thus forcing the gas into the cecum, which gas in turn forces the fecal mass upward, lodging it in the folds of the colon above. It has been said that the appendix is a useless member, that people live as well and as healthful after its amputation as they did before it; this may be true, but the cecum which is composed of similar tissue increases its muscular power and glandular function in proportion to the increased work; it thus compensates in a measure for the loss of the appendix. From a mechanical standpoint the curvature of the appendix, by a fibrous band, has its value, for the contraction of the muscular fibers of this organ gives to the wall on the convex side a lifting as well as a lateral motion without materially impairing its function as a reservoir for the accumulated gases, while favoring materially the elimination of the exudate from the solitary glands.

As to the etiology of appendicitis little can be said. The disease may be caused by traumatism, by constipation, by indiscretions in diet, by the entrance of a foreign body into the canal, by fecal concretions, and yet at other times inflammatory action has been induced with the conditions just stated wanting; it is possible then, and to me seems probable that such inflammatory action, as a rule, is induced by the activity and development of some specific microörganisms within the appendix and that the vitiated exudate from the solitary glands therein forming the menstruum in which the reproduction of such organisms take place.

For purposes of treatment two forms of appendicitis may be recognised—namely, catarrhal and ulcerative; but to my mind the primary cause of the inflammatory action in each case is the growth and reproduction of specific microörganisms within the cecum and appendix, and that the fecal masses, or other inanimate foreign bodies, within the appendix are coincidences not causes of the pathological changes. It will be observed that nature endeavors to limit in extent all exudations, whether mucoid or purulent, by producing thickening and adhesions of the inflamed parts to the surrounding tissues. Such adhesions usually precede pus formation, thus forming a circumscribed abscess. Such abscesses will point somewhere and that will be toward the line of least resistance, and that line extends toward the cecum when that organ is free from fecal accumulation.

The indications for treatment then are two-fold:

First.—To limit in extent or check entirely inflammatory action and prevent, as far as possible, mucoid and especially pus formation.

Second.—To eliminate all foreign substances, whether animate or inanimate, that produce directly or indirectly pathological changes.

The methods of treatment of appendicitis are various and they correspond to the individual ideas of the attending physician, such ideas being based upon individual observation or upon information obtained from "authorities." The treatment in vogue may be classed as surgical, medical and medico-surgical.

Several years ago before a differential diagnosis had been established and the action known as localised peritonitis, opium was the sheet-anchor; it must be given in heroic doses. The drug did doubtless prolong life and relieve pain and possibly checked pus formation, but it caused the fecal concretions, pus, etc., to remain within their bed and keep active, though in less degree, the pathological changes until death ended all. It used to be said "opium is our sheet anchor, but the patient will probably die," a confession of its worthlessness, save as an agent to relieve pain. After a differential diagnosis had become established opium began to go into disuse, though some practitioners still cling tenaciously to it and prescribe it in heroic doses. This drug does produce an apparent improvement, but the final results are more disastrous because of obscured conditions due to opium.

Following the advent of a differential diagnosis the pendulum swung to the surgical side and unfortunate was that practitioner who dared question its efficacy. Appendicitis became a by-word, its mention meant an operation; moreover when unmentionable because of its absence, such operations were advised even upon healthful babies, because, it was alleged, the appendix was of no use. But the pendulum is swinging back and soon surgical intervention will assume its true relation with other agencies in dealing with this malady. Surgical intervention erred in that it assumed to be the treatment while it was only auxiliary. The removal of the appendix with the accumulated débris is not enough; the cecum and colon should be cleansed of their accumulation prior to surgical interference in order that further accumulation may be readily passed off through the intestinal canal. Preparation before surgical intervention therefore is essential.

But if surgical intervention be called for and must precede the preparation, then clear the cecum and ascending colon of their contents by manipulation before closing the wound, thus giving the remaining and yet-to-be-formed débris a chance to escape.

Whether the existing cause of appendicitis be fecal accumulations, foreign bodies or accumulated excreta from the glands of the appendix matters but little so far as rational treatment is concerned; the object sought is the elimination from the diseased tissues all of the sources of irritation of every nature. Individual judgment must decide in each individual case the proper remedies and proper methods of treatment. External applications as leeches, cold compresses, poultices, blisters and similar procedures over the cecal region, as recommended by some, and still used by many, have no place in rational therapeutics; this statement after a moment's reflection must appear axiomatic. The first condition of successful treatment is absolute rest, and this condition excludes active catharsis, connected with which comes the danger of perforation of the diseased tissues.

Bearing in mind the pathology of appendicitis, bearing in mind the fact that nature does much toward repair and restoration of the infected parts, the treatment then becomes very much simplified. In accordance with this idea a treatment has been devised which has produced most favorable results. The cleaning out of the intestinal canal nature demands, and to see that it is done and done early and well is the duty of the physician, and for this purpose a personal supervision of the patient and the work is essential; a work that requires thoroughness and gentleness. This part of the treatment may be well accomplished by the use of the long tube which may be a soft rubber one, or a metallic one with the proper curvatures. The tube must be passed upward at least to the angle formed by the juncture of the transverse and descending colon; the patient to recline upon the right side with the hips well elevated; position here is essential and the reason for it is very apparent.

By means of the long tube the organs just mentioned can be cleansed by filling with water, impregnated with some good antiseptic material, preferably creolin or permanganate of potassium. Antiseptics are necessary, for following an attack of appendicitis, however mild, the intestinal canal is in a septic condition and from this there may be general systemic infection independent of the diseased appendicular tissues. This process of lavage may be repeated several times daily if necessary, as the severity of the case demands without producing material harm, depressing the vital forces, or interfering with the reparative processes. Thoroughness is essential to success.

Having first thoroughly cleansed the colon by lavage, then attention should be directed to the emptying of the upper portion of the alimentary canal; that the lower portion needs first attention is obvious, for a forcible ejection beginning from above increases the tension below and increases the liability to rupture of the affected part.

The entire canal must be kept scrupulously clean during the active stage of the affection which activity is limited as to duration; for this purpose solid foods, so-called, must be prohibited and it is questionable if even liquid foods are advisable. What is a food in a normal condition is quite likely to become an irritant, if not a poison, in an abnormal condition of the digestive apparatus; nourishment may be a good thing, but it must be administered with discretion.

Since the body is composed largely of water, since it requires no labor on the part of the digestive apparatus to absorb it when taken, since life may be supported for days by this element alone, why withhold it? It serves two most excellent purposes, viz.: that of food and also as a flushing and cleansing agent. For obvious reasons the normal saline solution might be preferred and substituted for water with advantage.

Of the various agents that have been used and served a good purpose, first, the oils, both castor and olive, rank high; they may be given alternately or combined; their action is largely a mechanical one, serving to lubricate and dissolve the intestinal contents; and they also form a barrier along the intestinal wall preventing, in some degree, the absorption of septic matter. Second, as a digestant and solvent, and perhaps as an intestinal stimulant, purified pig's gall ranks among the first and its use from a physiological standpoint, is clearly indicated in all catarrhal affections of the intestinal tract, including appendicitis. It being one of the normal agents in the process of digestion and having some antiseptic properties it can do no harm, but, on the other hand, it must be an agent for good. If pig's gall softens the fecal matter, if it stimulate to activity, without fatigue, the muscular tissue of the bowel, if it be an antiseptic that prevents or retards germ development, and these properties this agent is alleged to have, its uses in appendicitis is certainly suggestive and practically it has been found equal to the suggestion.

Agents other than those which have been enumerated may have their merits, may have their advantages even, and the selecting of them must be left to the individual judgment of the prescriber. The routes for attaining the same goal may be various, but the goal sought for in each case is the same—namely, complete elimination of all intestinal contents, whether normal or abnormal, including the products of inflammatory action about the cecum.

It is impossible, however, to secure perfect elimination of all intestinal contents at once; intestinal antiseptics are therefore suggested and of the number used sulphocarbolate of soda combined with bichloride of mercury is preferred. The line of treatment cannot be a fixed or routine one, it must be varied to meet the varying pathological conditions, and unfortunate must he be, as well as the unfortunate patient, who endeavors to treat appendicitis by name and fails to recognise preëxisting conditions.

In the earlier stages, prior to pus formation, the treatment is plain; it must be prophylactic, consisting of intestinal cleansing, followed by intestinal rest; but after pus formation a decision must be made between the medical, the surgical or the medico-surgical treatments, a decision, the reaching of which costs the country practitioner no little anxiety.

The accumulated débris in and about the appendix must be eliminated, and the "how" is the great problem for solution. In solving this problem the fact must be recognised that the physician's or surgeon's manipulations must be an aid to eliminative processes only, and such manipulations must be in harmony with nature's methods and such eliminative methods are in no wise connected with reparative processes.

Nature's route for the elimination of débris seems to be by way of the intestinal canal. Clear the cecum and colon of all fecal accumulations and nature will, in a majority of cases, care for the rest. "Authorities" must not be followed like sheep that follow the "bell-wether;" individual action and judgment must enter into each individual case. Commonsense methods must dictate, must rule, then shall we have fewer "successful operations" that end in successful deaths.

22 East Miller Street.

